# Image Similarity Judgment Method for Waste Printed Circuit Boards

**DOI:** 10.3390/s26041224

**Published:** 2026-02-13

**Authors:** Hikaru Shirai, Ryo Oishi, Yoichi Kageyama, Kazune Sasaki, Keita Ogawa, Satoshi Nakagawara

**Affiliations:** 1 Department of Informatics and Data Science, Faculty of Informatics and Data Science, Akita University (Tegata Campus), 1-1 Tegata Gakuen-machi, Akita-shi 010-8502, Akita, Japan; kageyama@ie.akita-u.ac.jp; 2 Department of Mathematical Science and Electrical-Electronic-Computer Engineering, Graduate School of Engineering Science, Akita University (Tegata Campus), 1-1 Tegata Gakuen-machi, Akita-shi 010-8502, Akita, Japan; 3 DOWA TECHNOLOGY CO., LTD., 71 Otarube, Kosaka-machi, Kazuno-gun, Akita 017-0202, Japan; 4 DOWA METALS & MINING CO., LTD., 60-1 Otarube, Kosaka-machi, Kazuno-gun, Akita 017-0202, Japan

**Keywords:** waste printed circuit boards (WPCBs), image processing, image similarity, complexity

## Abstract

Waste printed circuit boards (WPCBs) contain valuable metals such as gold, palladium, and silver, which are typically recovered through non-ferrous metal smelting. Currently, WPCBs are manually classified by workers, who visually compare board colors and component layouts with previously processed boards. This approach is time-consuming and prone to human error. To address these limitations, we propose an image-based algorithm for automated WPCB similarity assessment. The method extracts visual features from board images and computes similarity scores, incorporating classification strategies based on board-specific characteristics. Key features identified as effective for similarity evaluation include the hue value, coefficient of variation in terminal regions, number of line elements in terminal regions, structural complexity, and number of integrated circuits. Weighted feature contributions further improve accuracy. Our experimental results demonstrate that the proposed approach achieves 88.0% accuracy for the targeted PCB types, outperforming a comparative self-supervised contrastive learning method. This image-driven solution can significantly streamline WPCB recycling by reducing reliance on manual inspection and improving operational efficiency.

## 1. Introduction

In Japan, the working-age population has been declining in recent decades, and electronic waste (e-waste) has emerged as one of the most pressing environmental challenges of the 21st century. According to recent reports, the amount of global e-waste reached 53.6 million metric tons in 2019, and projections indicate that this figure will increase to approximately 75 million metric tons by 2030 if effective mitigation strategies are not implemented [1]. This alarming trend underscores the urgent need for sustainable e-waste management practices. Among the various components of e-waste, waste printed circuit boards (WPCBs) are particularly critical, owing to their complex composition and high economic value. WPCBs are integral components of a wide range of electronic products, including personal computers, smartphones, televisions, and household appliances. They contain a diverse array of materials, including polymers, ceramics, and metals, as well as precious and rare metals such as gold, palladium, and silver [2]. Consequently, WPCBs are often referred to as “urban mines”, signifying their potential as a sustainable source of valuable resources if properly managed [3]. However, the current state of WPCB recycling is far from optimal. Despite the economic incentives associated with metal recovery, only 17.4% of global e-waste was recycled effectively in 2019, with the remainder either improperly disposed of or exported to developing countries where environmental regulations are less stringent [4]. This practice not only exacerbates ecological degradation but also poses significant health risks to local communities. The improper handling of WPCBs can lead to the release of hazardous substances such as lead, mercury, and brominated flame retardants into the environment, contaminating soil, water, and air [5]. In addition to environmental and health concerns, inefficient recycling of WPCBs represents a substantial economic loss, as valuable metals remain unrecovered. To address these challenges, numerous countries have enforced regulations designed to promote the recycling of e-waste and the recovery of resources. For instance, the European Union’s Waste Electrical and Electronic Equipment (WEEE) Directive mandates the collection and recycling of electronic waste, while similar initiatives have been introduced in other regions [6]. Nevertheless, the implementation of these policies has been uneven, and significant gaps persist in the global e-waste management framework. In this context, the development of innovative technologies for WPCB recycling is crucial in enhancing resource efficiency, mitigating environmental impact, and facilitating the transition toward a circular economy. Recent systematic reviews have emphasized that artificial intelligence-based approaches are becoming increasingly important in improving the efficiency and sustainability of WPCB recycling processes [7].

The recycling of WPCBs typically involves a series of processes designed to recover valuable metals and minimize environmental harm. One common approach is pyrometallurgical processing in non-ferrous metal smelters, where WPCBs are subjected to high temperatures to extract metals such as gold, palladium, and silver [8]. While effective in terms of metal recovery, this method is energy-intensive and can generate harmful emissions if not adequately controlled. An alternative approach is hydrometallurgical processing, which employs chemical leaching to dissolve metals from WPCBs. Although less energy-intensive, this method requires the use of hazardous chemicals, raising concerns about secondary pollution [9]. Regardless of the specific recycling technique employed, the initial step in the process often involves the visual inspection and classification of WPCBs. In many recycling facilities, images of the target “lot” of the printed circuit boards are captured, followed by compositional analysis through crushing and sampling of the material. The measured metal content ratios are then compared against a database to identify records with similar compositions. Subsequently, printed circuit board (PCB) images from matched records are manually compared with those of the target lot to assess visual similarity based on attributes such as the color, size, and component layout [10]. If the images are deemed similar, then the compositional analysis results are approved for processing; otherwise, they are re-evaluated. This workflow is highly subjective, relying on the judgment of operators without standardized quantitative metrics. Recent advances in deep learning-based PCB inspection have demonstrated that convolutional autoencoder architectures can effectively detect subtle defects and structural variations on circuit boards, offering a more objective alternative to manual visual assessment [11]. Semi-supervised learning approaches have also been shown to achieve high defect detection performance with limited labeled data, making them particularly suitable for large-scale PCB inspection scenarios where manual annotation is costly [12]. Moreover, the process is labor-intensive and time-consuming, particularly given the large volumes of WPCBs that must be evaluated in industrial recycling operations. The reliance on manual inspection not only increases operational costs but also introduces the risk of human error, which can compromise the accuracy of classification and subsequent processing decisions [13]. These limitations highlight the need for automation in WPCB recycling, particularly in the context of image-based similarity assessment. By leveraging advanced image processing technologies, it is possible to reduce dependence on manual inspection, enhance the consistency of evaluations, and improve overall process efficiency. Furthermore, recent analyses of deep learning-based PCB defect detection models have highlighted the importance of optimized training strategies to ensure robust and reliable automated inspection performance in practical applications [14].

Image similarity measurement has been extensively studied in various domains, including computer vision, pattern recognition, and multimedia retrieval. Traditional approaches to image similarity assessment often rely on feature-based methods, such as Scale-Invariant Feature Transform, Speeded-Up Robust Features, and Oriented FAST and Rotated BRIEF, which extract local descriptors from images and compute the similarity based on feature matching [15,16,17,18]. While these methods have demonstrated robustness to certain variations in scale and rotation, they are sensitive to changes in illumination and occlusion, which are common in industrial environments. In recent years, deep learning techniques have revolutionized image analysis, offering superior performance in tasks such as object recognition, image classification, and similarity measurement. Convolutional neural networks, in particular, have been widely adopted for feature extraction and representation learning, enabling the development of end-to-end systems for image retrieval and matching [19,20,21,22,23,24,25,26,27]. Advanced architectures, such as Siamese Networks and Vision Transformers, have further enhanced the capability of deep learning models to capture complex visual patterns and compute similarity with high accuracy. In the context of PCB analysis, several studies have explored the application of image processing and machine learning techniques to automate tasks such as defect detection, component recognition, and classification. Firsching et al. proposed a method for estimating component composition by detecting the parts in X-ray images of PCBs using YOLOv5, a state-of-the-art object detection algorithm [28]. Iftikhar et al. introduced a robust PCB classification system based on deep convolutional neural networks, demonstrating significant improvements in accuracy compared to those in traditional methods [29]. Mohsin et al. proposed a method for identifying components in waste electronic circuit boards using YOLOv10 [30]. Christopher et al. employed local feature matching techniques for PCB recognition, while Vilas et al. developed an efficient similarity measurement approach for detecting surface defects on PCBs [31]. Vilas et al. also developed an efficient method for measuring similarity and detecting surface defects on PCBs [16]. Silva et al. proposed a technique for estimating PCB values by extracting integrated circuits (ICs) from PCB images and analyzing their surface area [32]. Despite these advancements, existing studies primarily focus on individual PCB images captured in controlled settings. In contrast, recycling facilities often process large quantities of PCBs simultaneously on conveyor belts, resulting in images that contain multiple boards with varying orientations, overlaps, and lighting conditions. This operational context introduces additional complexity that conventional methods do not adequately address. Furthermore, most existing approaches do not incorporate domain-specific features relevant to WPCBs, such as the terminal area characteristics and IC density, which can significantly influence similarity assessments. These limitations highlight the need for specialized algorithms that can effectively handle the unique challenges inherent in WPCB image analysis within industrial recycling environments.

To bridge the gap identified in the existing literature, in this study, we aimed to develop a novel algorithm for automating the determination of similarity in WPCBs using image-based analysis. The proposed method calculates image features and similarity scores from PCB images and evaluates the classification accuracy based on PCB characteristics. Unlike previous studies that relied on generic image descriptors or deep learning models trained on unrelated datasets, our approach incorporates domain-specific features tailored to the structural and compositional attributes of WPCBs. Specifically, the algorithm considers five key image features: (1) hue values, (2) coefficient of variation in terminal areas, (3) number of lines in terminal areas, (4) complexity of the PCB area, and (5) number of ICs. By applying optimized coefficients for each PCB type, the method enhances the accuracy of similarity determination, achieving a success rate of 88.0% in experimental evaluations. This performance represents a substantial improvement over manual inspection and existing automated techniques, demonstrating the efficacy of the proposed approach in real-world recycling scenarios. The contributions of this study are threefold. Firstly, it introduces a comprehensive framework for assessing the image-based similarity of WPCBs, addressing limitations in current practices. Secondly, it provides empirical evidence of the effectiveness of domain-specific feature selection and weighting in improving the similarity measurement accuracy. Thirdly, it highlights the practical implications of automation in WPCB recycling, including reduced operational costs, enhanced process efficiency, and minimized human error. By advancing the state of the art in WPCB image analysis, this research supports the broader goal of sustainable e-waste management and contributes to the development of technologies that facilitate the transition toward a circular economy. The remainder of this paper is organized as follows: Section 2 describes the dataset and PCB types analyzed, Section 3 details the proposed similarity calculation method, Section 4 presents the experimental results, and Section 5 concludes the study.

A survey of the existing literature reveals that a large number of studies have been devoted to PCB analysis and inspection, including deep learning-based defect detection, component recognition, and classification methods, as well as image similarity and retrieval approaches developed for controlled laboratory environments. In this study, more than 30 related articles were reviewed, and they are cited throughout Section 1.

Despite these advances, several research gaps remain in the context of industrial waste printed circuit board (WPCB) recycling. Firstly, most state-of-the-art methods rely on learned image representations and require large amounts of labeled data, which are often difficult to obtain in recycling facilities. Secondly, existing similarity assessment approaches typically operate on individual board images and do not account for lot-level variation encountered in real processing workflows. Thirdly, the interpretability of similarity judgments, which is critical in practical decision-making by operators, is rarely addressed.

These gaps justify the objective of this study: to develop a training-free, interpretable image similarity judgment method that integrates domain-specific visual and structural features and operates at the lot level under realistic industrial conditions. It should be emphasized that the novelty of this study does not stem from the development of new image descriptors or learning models. Rather, its contribution lies in formulating a domain-specific image similarity judgment framework for WPCB recycling, in which existing and interpretable image features are systematically integrated, weighted according to PCB type, and applied at the lot level to support practical recycling operations.

## 2. Data Used

In this paper, the term “electronic circuit board” refers to a PCB equipped with multiple ICs and electronic components. A PCB is designed to mount electronic components and interconnect them through wiring patterns of thin copper foil on an insulating substrate [33]. PCBs typically use epoxy resin, a type of plastic, as the base material. Depending on the application, other materials, such as Bakelite or flexible PCBs, which comprise thin plastic films with printed copper foil, can also be used. Thus, PCBs contain economically valuable metals such as gold and copper.

Circuit board images are categorized based on factors such as the date of capture and board type and are managed in units called “lots”. A lot refers to a large batch of circuit boards handled as a single unit, typically on the order of several tons. Circuit boards are fed onto a belt conveyor on a lot-by-lot basis, during which images are captured for analysis. Each image includes multiple circuit boards simultaneously; several boards appear within a single frame. Depending on the total quantity of boards in a lot, multiple images are acquired for the same lot. As a result, one lot is associated with a set of images, all of which represent different portions of the same batch of circuit boards processed under identical conditions. The dataset for the circuit boards includes the board type, lot number, and metal content ratio. The metal content ratio represents the proportion of valuable metals, such as gold, silver, and palladium, contained in the circuit boards within a single lot, expressed in parts per million (ppm). This value corresponds to the aggregated metal content of the entire lot rather than individual boards.

**Figure 1:**
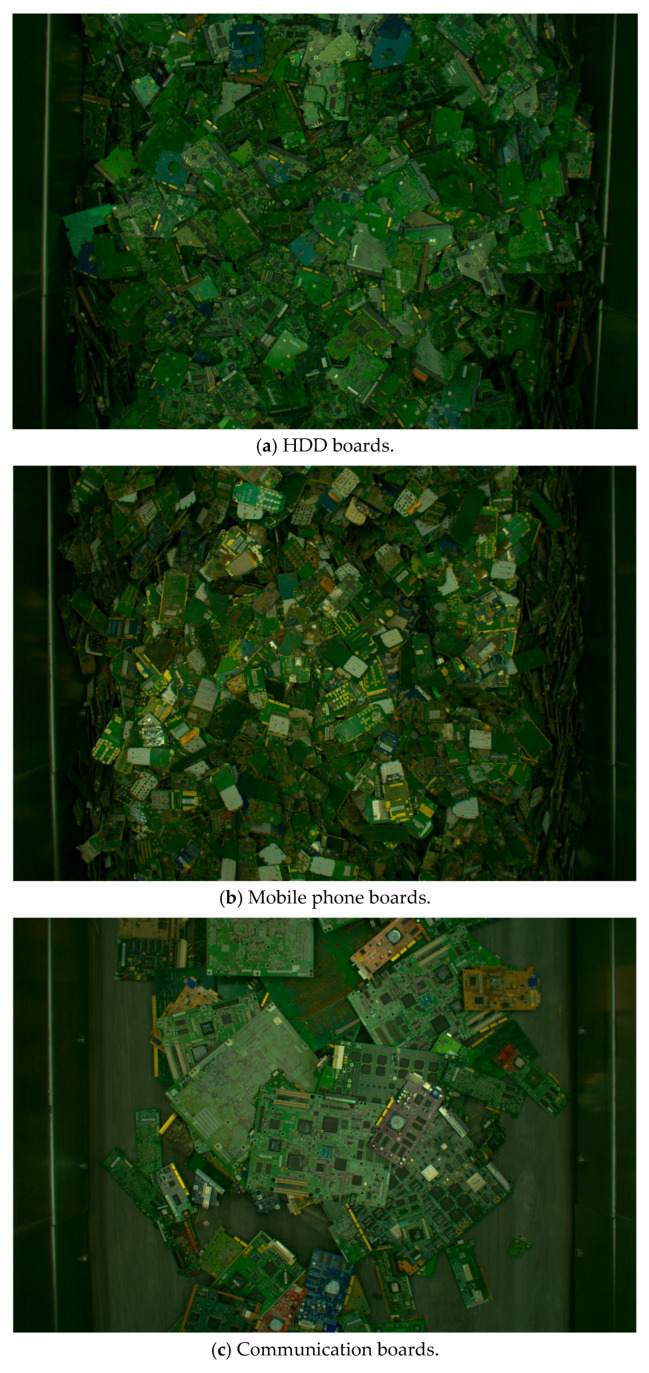

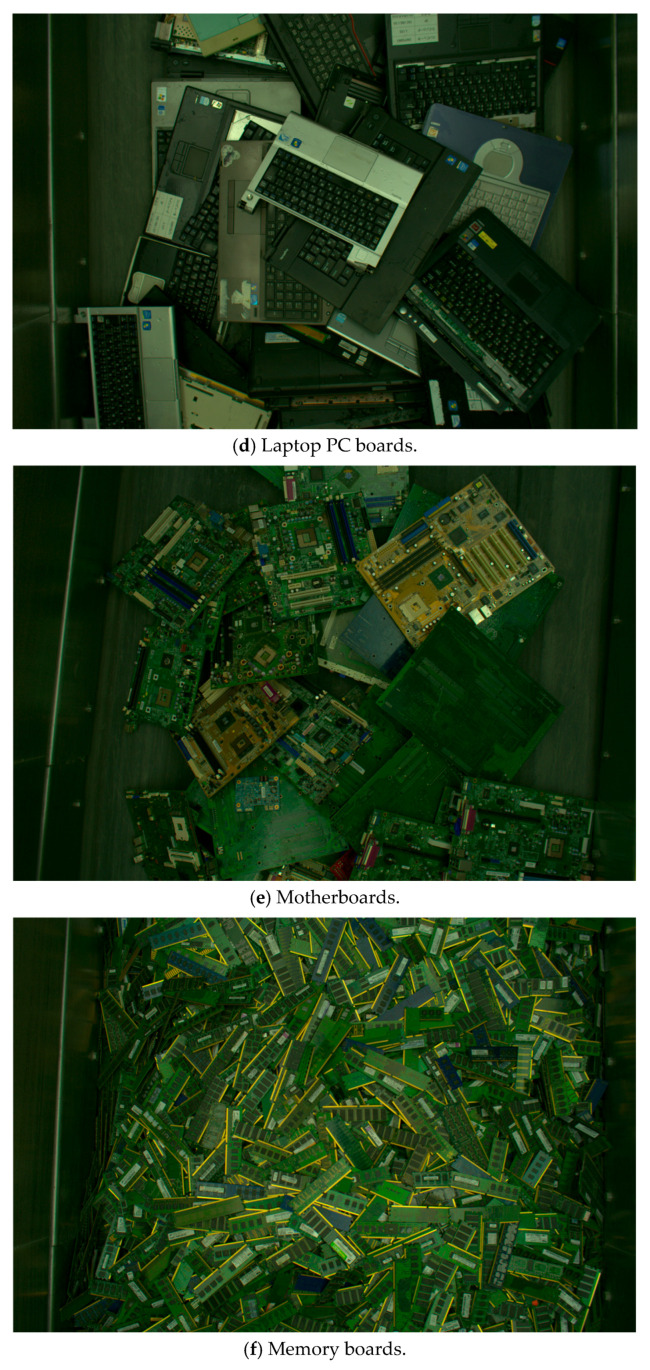

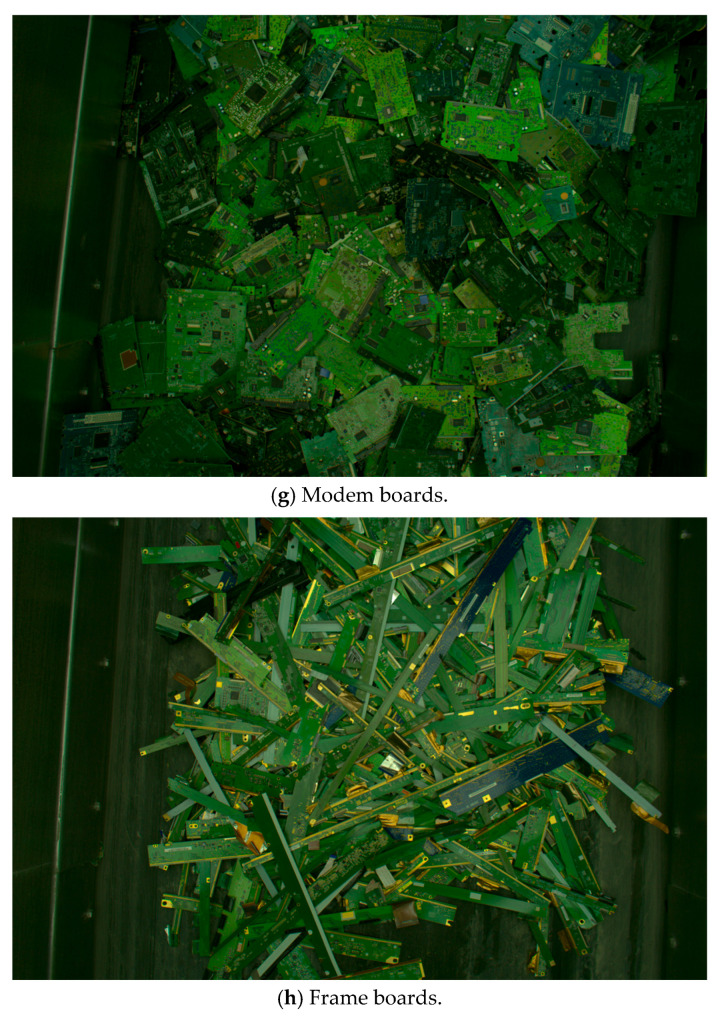
Examples of circuit board images used in this study.

In this study, 10 types of circuit boards with a large number of lots and a wide range of gold content ratios were selected as the target boards. These types included hard disk drives (HDDs), mobile phones, communication devices, laptop PCs, motherboards, memory, modems, and frame boards. Table 1 presents the number of lots for each type. Figure 1 shows representative sample images from the Industrial WPCB Lot Image Dataset used in this study.

In the industrial workflow considered in this study, PCB lots are already categorized by board type prior to image analysis. The focus of this research is not on PCB type classification but on similarity judgment among lots within the same PCB category. Even within a single PCB type, significant variations in visual appearance, structural characteristics, and metal content can be observed, making manual similarity assessment time-consuming and subjective.

**Figure 2:**
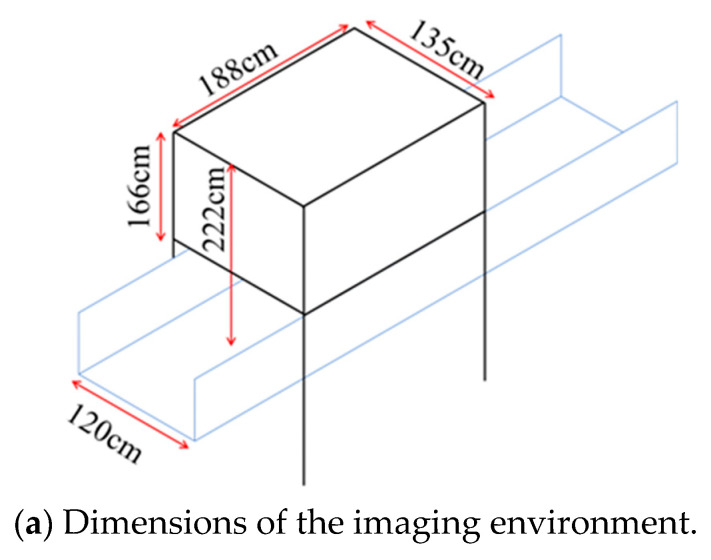

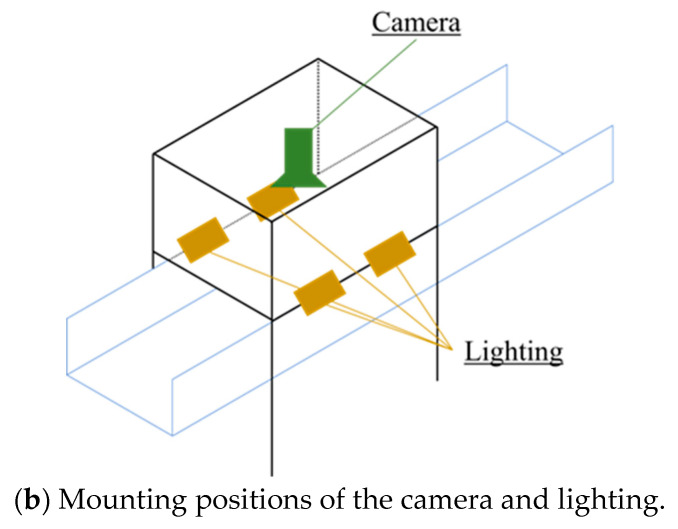
Overview of the imaging environment. A dark chamber was installed above the conveyor belt, and four lighting units were placed inside it. The camera was mounted at the center of the top of the chamber.

All PCB images used in this study were captured in a controlled darkroom environment, in which external light was completely blocked. The camera system and illumination setup were fixed, and images were acquired under consistent lighting and camera settings throughout the data collection process. Owing to this controlled imaging condition, the influence of ambient light fluctuations and camera-related variability was minimized, and strict color calibration procedures were not considered necessary. An overview of the imaging environment is shown in Figure 2, and the specifications of the camera used are summarized in Table 2.

## 3. Proposed Method

### 3.1. Overview of the Proposed Method

**Figure 3:**
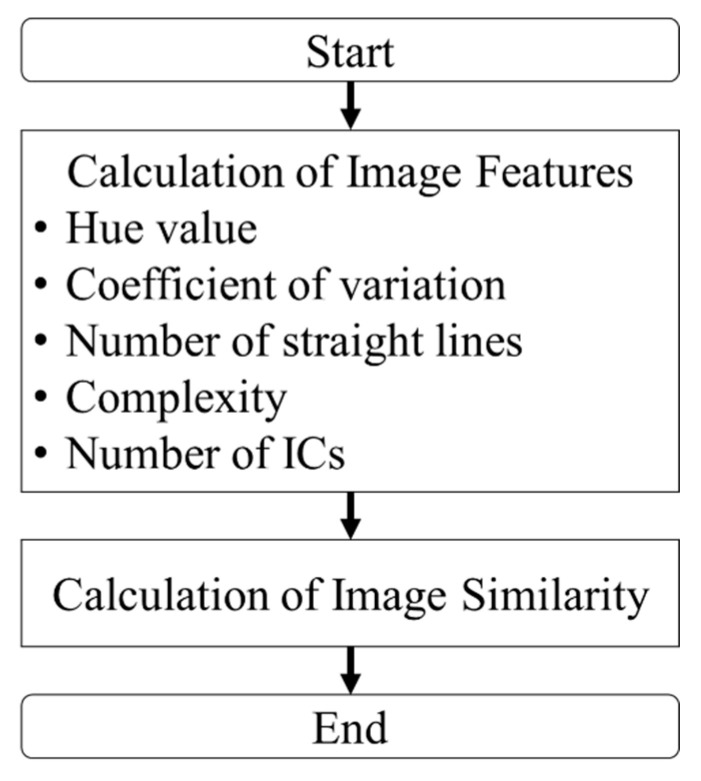
Overview of similarity assessment method using circuit board images in this study.

Figure 3 presents an overview of the similarity assessment method. Here, the input lot represents the lot to be evaluated for similarity, whereas the database refers to the target lots for comparison within the circuit board dataset. First, image features are extracted from the input lot using parameters such as the hue value [34], coefficient of variation [35], number of straight lines [36], complexity [37], and number of ICs. Subsequently, the similarity between images is calculated based on the extracted image features. Although the overall structure of the workflow in Figure 3 follows a general feature extraction-based similarity assessment framework, the key innovation lies in the domain-specific design and integration of each processing step. The proposed method explicitly incorporates multiple interpretable features that capture the color, structural complexity, terminal distribution, and component density of waste printed circuit boards. In addition, similarity is evaluated at the lot level, reflecting actual industrial recycling operations in which multiple boards are processed simultaneously. Furthermore, feature contribution weights are optimized according to PCB type, enabling flexible adaptation to product-specific characteristics without requiring a training phase. These aspects distinguish the proposed framework from conventional image similarity pipelines and learning-based approaches. Accordingly, the proposed method should be understood as a domain-orientated similarity judgment framework rather than a contribution based on novel image features or learning algorithms. The emphasis is on adapting existing image analysis techniques to the specific requirements of WPCB recycling, including interpretability, lot-level evaluation, and robustness under industrial constraints.

The proposed method is designed to retrieve visually and structurally similar PCB lots from a database of the same PCB type. It is not designed to distinguish between different PCB categories; rather, it supports similarity-based retrieval within a predefined PCB type to assist in industrial recycling operations.

For clarity and readability, the complete step-by-step algorithmic procedure of the proposed method is provided in Appendix A (Algorithm A1).

### 3.2. Calculation of Image Features

Automating similarity assessment in circuit board images requires the image similarity to be expressed in numerical terms. In this study, similarity scores were calculated based on image information for any two lots (i.e., input lot and database lot) of the circuit board images.

The procedure for calculating image features is as follows. First, hue (H) component features in the HSV color space are extracted from circuit board images of both the input and database lots. Second, features based on the coefficient of variation are computed, followed by the extraction of the number of straight lines. Subsequently, structural complexity features are extracted, and finally, the number of ICs is determined. These features are then used to compute the image similarity.

#### 3.2.1. Features Related to Hue Values

The types of colors contained in circuit board images vary depending on the board type; consequently, hue values are useful features for assessing image similarity. Therefore, features were calculated based on the hue values. Specifically, all pixels in the circuit board images of the input and database lots were classified according to their hue values (hue classification), and the proportion of pixels in each hue category was calculated. Next, features related to the hue values were extracted.

The HSV color space represents colors using three components, namely, hue (H), saturation (S), and value (V). H is expressed as a numerical value from 0 to 360, whereas S and V are represented within a range of 0 to 100. Compared with the RGB color space, the HSV color space can capture color differences in a manner closely aligned with human perception [38]. Hence, hue-based features can be used to automate similarity assessment in circuit board images, a process that is conventionally performed through visual inspection. HSV components can be derived from RGB components using a conversion formula [34]. Hue values obtained from circuit board images can fluctuate because of variations in the imaging environment, resulting in discrepancies between the captured and actual colors of the boards. To mitigate this discrepancy, the hue values were classified into 24 categories (hue classifications), and the hue values of adjacent classifications were set to overlap by five units. Table 3 presents the correspondence between the hue classifications and hue values.

For any two lots (i.e., input lot and database lot), the hue value of each pixel was extracted, and each pixel was assigned the corresponding hue classification based on this value.

Firstly, using Equations (1) and (2), the ratio of the number of pixels in each hue classification to the total number of pixels was calculated for the circuit board images in both the input lot and the database.

(1) $$f_{c_{i}}=\frac{A_{c_{i}}}{N_{A}}\left(i:1–24\right)$$

(2) $$f_{{cd}_{i}}=\frac{B_{c_{i}}}{N_{B}}\;\left(i:1–24\right)$$

Here,

i: Hue category;
$f_{c_{i}}$: Ratio of pixels in the *i*-th hue category of the input lot image;
$f_{{cd}_{i}}$: Ratio of pixels in the *i*-th hue category of the database image;
$A_{c_{i}}$: Number of pixels in the *i*-th hue category of the input lot image;
$B_{c_{i}}$: Number of pixels in the *i*-th hue category of the database image;
$N_{A}$: Total number of pixels in the input lot image;
$N_{B}$: Total number of pixels in the database image.

Finally, feature $F_{C}$ was calculated for the hue values using the following expression:

(3) $$F_{c}=\frac{1}{24}\sum_{i=1}^{24}\left(1-\frac{\left|f_{c_{i}}-f_{{cd}_{i}}\right|}{f_{{cdi}_{max}}-f_{{cdi}_{min}}}\right)$$

Here,

$f_{{cdi}_{max}}$: Maximum ratio of pixels in the *i*-th hue category of the database image;
$f_{{cdi}_{min}}$: Minimum ratio of pixels in the *i*-th hue category of the database image.

#### 3.2.2. Features Related to the Coefficient of Variation in the Terminal Domain

The distribution of terminal sections in circuit board images varies depending on the type of board. Therefore, quantifying these distribution differences is useful for board classification. To achieve this quantification, the coefficient of variation for the images was calculated based on the pixels corresponding to the terminal sections. Next, the coefficient of variation obtained from the circuit board images in both the input lot and the database was used to compute the feature values.

Circuit board images contain various components in addition to terminal sections. Therefore, because the focus is on terminal sections, a mask image was created to extract only the terminal sections. Specifically, pixels corresponding to the range presented in Table 4 were set as white pixels (representing terminal sections), whereas all other pixels were set as black pixels.

**Figure 4:**
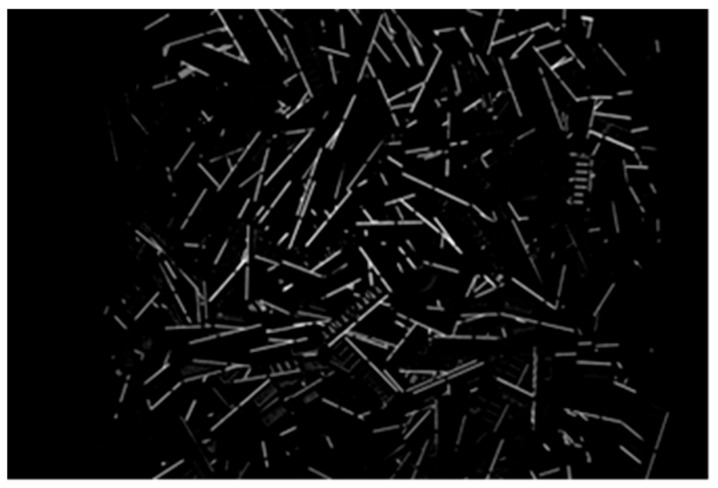
Example of a mask image for terminal sections (memory boards).

First, the regions that satisfy the HSV component ranges listed in Table 4 were extracted as areas corresponding to terminal sections. Figure 4 depicts an example of a terminal mask image. Each HSV component was determined by sampling the terminal sections.

**Figure 5:**
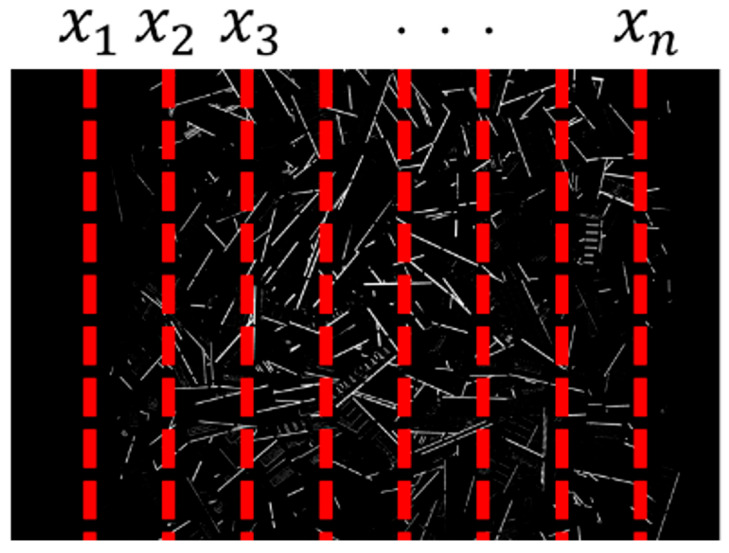
Example of a perpendicular line configuration.

Second, vertical lines were placed at 5-pixel intervals from the left edge of the terminal section mask image of each lot (Figure 5), and the total number of white pixels along each vertical line was calculated.

**Figure 6:**
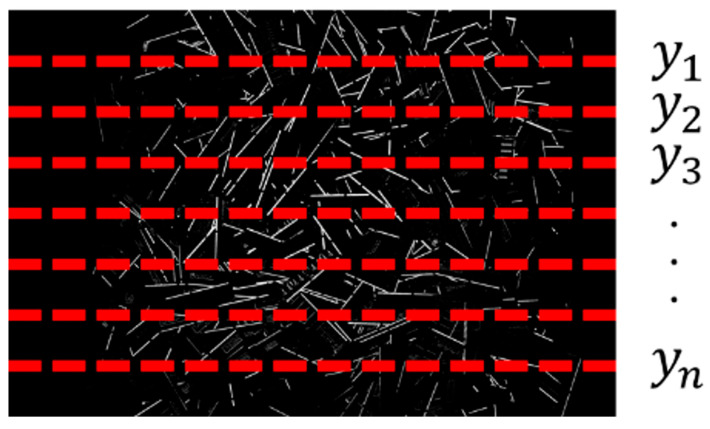
Example of a parallel line configuration.

Third, horizontal lines were placed at 5-pixel intervals from the top edge (Figure 6), and the total number of white pixels along each horizontal line was calculated. The perpendicular and parallel line configurations shown in Figure 5 and Figure 6 are introduced to characterize the spatial distribution of terminal regions. In practical PCB designs, terminals and connectors are often arranged along specific edges or directions depending on interface requirements. By evaluating the distribution of terminal pixels along vertical and horizontal directions, these configurations capture differences in terminal density, alignment, and layout regularity, which reflect physical design characteristics of PCBs.

Fourth, the coefficient of variation was calculated using Equations (4) and (5):

(4) $$f_{v}=\frac{\sqrt{\frac{1}{n_{x}}\sum_{k=1}^{n_{x}}{\left(x_{k}-\bar{x}\right)}^{2}}}{\bar{x}}+\frac{\sqrt{\frac{1}{n_{y}}\sum_{k=1}^{n_{y}}{\left(y_{k}-\bar{y}\right)}^{2}}}{\bar{y}}$$

(5) $$f_{vd}=\frac{\sqrt{\frac{1}{n_{x}}\sum_{k=1}^{n_{x}}{\left(x_{k}-\bar{x}\right)}^{2}}}{\bar{x}}+\frac{\sqrt{\frac{1}{n_{y}}\sum_{k=1}^{n_{y}}{\left(y_{k}-\bar{y}\right)}^{2}}}{\bar{y}}$$

Here,

$f_{v}$: Coefficient of variation in the input lot image;
$f_{vd}$: Coefficient of variation in the database image;
$x_{k}$: Number of white pixels on the *k*-th vertical line;
$y_{k}$: Number of white pixels on the *k*-th horizontal line;
$\bar{x}$: Mean number of white pixels on all vertical lines;
$\bar{y}$: Mean number of white pixels on all horizontal lines;
$n_{x}$: Number of vertical lines;
$n_{y}$: Number of horizontal lines.

Finally, feature $F_{v}$ was calculated for the coefficient of variation using the following equation:

(6) $$F_{v}=1-\frac{\left|f_{v}-f_{vd}\right|}{f_{{vd}_{max}}-f_{{vd}_{min}}}$$

Here,

$f_{{vd}_{max}}$: Maximum coefficient of variation in the database image;
$f_{{vd}_{min}}$: Minimum coefficient of variation in the database image.

#### 3.2.3. Features Related to Straight Lines

**Figure 7:**
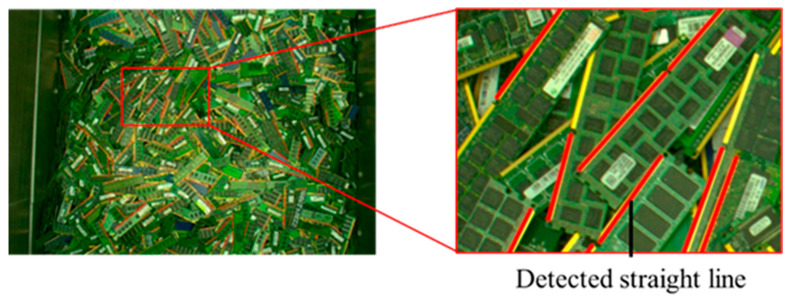
Example of detected line segments in the terminal-section (memory boards).

The number of terminal sections in a PCB image varies depending on the type of PCBs in each lot. Terminal sections are typically elongated; hence, detecting them as lines in a terminal section mask image enables their quantity to be estimated. Therefore, a line detection process was applied to the terminal section mask image, and the number of detected lines was used to compute a feature quantity related to the number of lines. The line detection process incorporates the terminal section mask image that was created during the computation of the coefficient of variation for the terminal sections. First, a line detection process [37] was applied to the terminal section mask image to determine the number of detected lines. Figure 7 depicts an example of the line detection result and its magnified result. Next, the feature quantity related to the number of lines $F_{L}$ was calculated using the following equation:

(7) $$F_{L}=1-\frac{\left|f_{L}-f_{Ld}\right|}{f_{{Ld}_{max}}-f_{{Ld}_{min}}}$$

Here,

$f_{L}$: Number of lines detected from the input lot image;
$f_{Ld}$: Number of lines detected from the database image;
$f_{{Ld}_{max}}$: Maximum number of lines detected from the database image;
$f_{{Ld}_{min}}$: Minimum number of lines detected from the database image.

The purpose of applying line detection in this study is to obtain a robust and interpretable feature that reflects relative differences in terminal density among PCB lots. In this context, false positive detections have a more detrimental effect on lot-level similarity than missed detections, because they directly inflate the feature value. Therefore, detection precision was intentionally prioritized over recall.

A quantitative evaluation using manually annotated ground-truth data yielded a precision of 0.998 and a recall of 0.498. The high precision indicates that false line detections are negligible, which is critical in maintaining stability of the line-count feature. The lower recall is mainly caused by terminal regions near image boundaries, where illumination is insufficient and terminal extraction itself becomes unreliable. Many of these missed terminals are also difficult to identify through visual inspection.

Because the detected line count is aggregated and normalized at the lot level and used only as one component of a multi-feature similarity framework, the observed recall level does not adversely affect the overall similarity judgment performance.

#### 3.2.4. Complexity Features

The size of the substrate in PCB images varies depending on the type of lot. Therefore, determining the distribution spread of the substrate region due to differences in lot types is considered useful for substrate classification. In this study, the complexity of the substrate region was examined using the inverse difference moment (IDM) [38], an index that decreases in value as the brightness difference between adjacent pixels increases. The value of the IDM ranges from 0.00 to 1.00.

First, the IDM was calculated using a gray-level co-occurrence matrix (GLCM). The GLCM is a statistical method used to examine the spatial relationship between pixels in an image. It quantifies the frequency of pairs of pixels with specific gray-level values that occur at a defined spatial relationship (distance and direction) within a given image. This calculation is as follows:

(8) $$\sum_{i=0}^{n-1}\sum_{j=0}^{n-1}\frac{1}{1+{\left(i-j\right)}^{2}}P_{\delta }\left(i,j\right)$$

Here, $P_{\delta }\left(i,\;j\right)$ is the GLCM.

**Figure 8:**
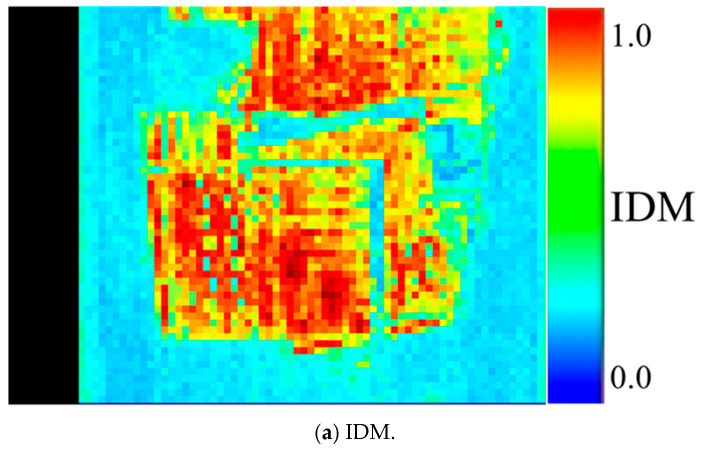

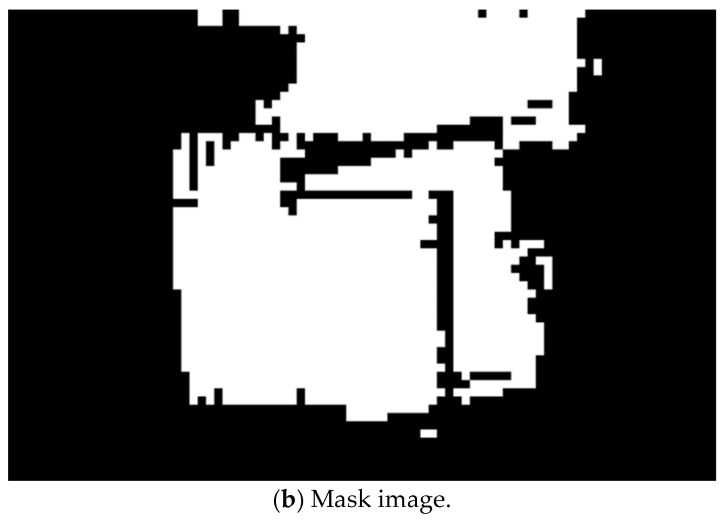
Example of a substrate region mask image used to validate the IDM-based extraction of the PCB substrate area for complexity feature calculation.

Second, threshold processing was applied to the calculated IDM to extract the substrate region and create a mask image for the substrate region. Figure 8 depicts an example of the extracted mask image for the substrate region. In this study, the appropriate value for accurately extracting the substrate region was examined, and areas with IDMs of 0.56 or higher were defined as the substrate region. The IDM threshold used for substrate region extraction was determined through a systematic parameter sweep. Specifically, the threshold value was varied from 0.01 to 1.00 in increments of 0.02, and the resulting substrate mask images were evaluated for their ability to consistently and accurately capture the PCB substrate region across different PCB types. Through this process, a threshold value of 0.56 was identified as providing the most stable substrate extraction for the dataset considered in this study and was therefore adopted. Although this threshold was effective for the controlled imaging conditions and PCB types examined, it is acknowledged that different PCB categories or imaging environments may require re-tuning of this parameter. Investigating adaptive or data-driven threshold selection strategies has been identified as an important direction for future work.

Third, the coefficient of variation for the substrate region mask image of each lot was calculated. Specifically, for the substrate region mask image of each lot, vertical lines were set at 5-pixel intervals from the left edge of the image, and the total number of white pixels on each vertical line was calculated. Similarly, horizontal lines were set at 5-pixel intervals from the top edge of the image, and the total number of white pixels on each horizontal line was calculated. Finally, the coefficient of variation was calculated using Equations (9) and (10):

(9) $$f_{s}=\frac{\sqrt{\frac{1}{n_{x}}\sum_{k=1}^{n_{x}}{\left(x_{k}-\bar{x}\right)}^{2}}}{\bar{x}}+\frac{\sqrt{\frac{1}{n_{y}}\sum_{k=1}^{n_{y}}{\left(y_{k}-\bar{y}\right)}^{2}}}{\bar{y}}$$

(10) $$f_{sd}=\frac{\sqrt{\frac{1}{n_{x}}\sum_{k=1}^{n_{x}}{\left(x_{k}-\bar{x}\right)}^{2}}}{\bar{x}}+\frac{\sqrt{\frac{1}{n_{y}}\sum_{k=1}^{n_{y}}{\left(y_{k}-\bar{y}\right)}^{2}}}{\bar{y}}$$

Here,

$f_{s}$: Coefficient of variation in the input lot image;
$f_{sd}$: Coefficient of variation in the database image;
$x_{k}$: Number of white pixels on the *k*-th vertical line;
$y_{k}$: Number of white pixels on the *k*-th horizontal line;
$\bar{x}$: Mean number of white pixels on all vertical lines;
$\bar{y}$: Mean number of white pixels on all horizontal lines;
$n_{x}$: Number of vertical lines;
$n_{y}$: Number of horizontal lines.

Finally, feature $F_{s}$ was calculated for the coefficient of variation using the following expression:

(11) $$F_{s}=1-\frac{\left|f_{s}-f_{sd}\right|}{f_{{sd}_{max}}-f_{{sd}_{min}}}$$

Here,

$f_{s}$: Number of lines detected from the input lot image;
$f_{sd}$: Number of lines detected from the database image;
$f_{{sd}_{max}}$: Maximum number of lines detected from the database image;
$f_{{sd}_{min}}$: Minimum number of lines detected from the database image.

The GLCM-based IDM quantifies the local uniformity of gray-level variations in the PCB substrate region. Boards with high wiring density and complex layouts tend to exhibit lower IDM values, whereas simpler and more uniform substrate regions result in higher values. This complexity measure is incorporated into the overall similarity score as one of the weighted components, enabling PCB lots with similar structural organization to be evaluated as more similar.

#### 3.2.5. Feature Quantity Related to the Number of ICs

The number of ICs mounted on a PCB varies depending on the type of PCB. The IC count is highly dependent on the structure and component layout of the board [39]. As a result, calculating the number of ICs enables a detailed characterization of each PCB. Moreover, as the IC count is correlated with the gold content ratio, the number of ICs could be an effective feature for determining PCB similarity.

In this study, a novel method for detecting ICs and determining their number was investigated using You Only Look Once (YOLO) [40], an object detection algorithm. YOLO is optimized for real-time object detection and simultaneously performs object presence recognition, class estimation, and bounding box prediction, enabling fast and accurate IC detection. Among its variants, YOLOv11 [40] has both improved detection accuracy and enhanced speed compared to previous YOLO models, making it suitable for high-precision IC detection. In this study, an IC counting model based on YOLOv11 was developed to automatically extract the number of ICs from PCB images.

In this process, a YOLOv11 model trained using annotated IC region data was applied to PCB images to detect ICs. The number of detected ICs was counted for each image, and based on these results, an IC count-based image feature was calculated to determine the image similarity.

First, during the annotation process, five images were randomly selected from each target PCB type that had exposed ICs. The regions corresponding to ICs in these images were manually annotated. During this process, different labels were assigned to the IC regions for each PCB type, considering the type of ICs present on each board.

Second, the model was trained using the annotated data. The training was conducted with the following parameter settings:

- Number of Epochs: 100;
- Batch Size: Automatically optimized;
- Mode: Training mode [40].

It should be noted that the IC detection model was trained using a limited number of manually annotated images (five images per PCB type). This design choice was made to minimize annotation cost and because the purpose of IC detection in this study was not precise component recognition but the extraction of a relative and interpretable feature for image similarity judgment within a fixed PCB type. Consequently, the IC count feature is used as an approximate descriptor of component density rather than an exact inventory of mounted ICs. While the current training data were sufficient to capture the dominant IC layouts for the targeted PCB types under controlled imaging conditions, expanding the number and diversity of annotated samples would further improve the robustness and generalizability of the IC detection model. These extensions have been identified as an important topic for future work. During inference, the total number of detected ICs was calculated for each PCB and used as a feature in the image similarity computation.

Third, using the trained YOLOv11 model, IC regions within the PCB images were detected. In this study, the number of regions enclosed by bounding boxes in the inference results was considered the IC count for the corresponding image.

Finally, feature $F_{I}$ related to the IC count was calculated using the following equation:

(12) $$F_{I}=1-\frac{\left|f_{I}-f_{Id}\right|}{f_{{Id}_{max}}-f_{{Id}_{min}}}$$

Here,

$f_{I}$: Number of ICs detected from the input lot image;
$f_{Id}$: Number of ICs detected from a database image;
$f_{{Id}_{max}}$: Maximum number of ICs detected among all database images;
$f_{{Id}_{min}}$: Minimum number of ICs detected among all database images.

### 3.3. Calculation of Image Similarity

The image statistics and parameters illustrated in Figure 3 correspond directly to the five image features defined in Section 3.2: the hue-based feature ($F_{c}$), terminal-region variation ($F_{v}$), terminal line feature ($F_{L}$), substrate complexity ($F_{s}$), and IC count feature ($F_{I}$). These feature values are computed for each PCB lot and integrated into the similarity score using the weighted formulation in Section 3.3. The experimental evaluation in Section 4 assesses similarity judgment performance based on these feature-derived similarity scores. The five feature quantities ($F_{c}$, $F_{v}$, $F_{L}$, $F_{s}$, and $F_{I}$) were calculated for the input lot and PCB images in the database. The degree of influence (contribution rate) of each feature quantity on image similarity determination varies depending on the type of substrate. For this reason, to achieve accurate similarity determination, one must consider the contribution rate of each feature quantity. Accordingly, coefficients α, β, γ, δ, and ε were assigned to each feature quantity, as presented in Equation (13). In this study, the values of α, β, γ, δ, and ε were set in increments of 0.1 within the range from 0.1 to 1.0, and the image similarity was calculated for 100,000 (10^5^) patterns:

(13) $$S=\frac{\alpha F_{c}+\beta F_{v}+\gamma F_{L}+\delta F_{s}+\varepsilon F_{I}}{\alpha +\beta +\gamma +\delta +\varepsilon }.$$

The exhaustive search used to determine the weighting coefficients is feasible because the parameter space is low-dimensional and discretized with a finite resolution. In this study, the coefficients are optimized offline for each PCB type and do not affect the runtime performance of the similarity judgment. Moreover, the evaluation criterion used for optimization is non-differentiable, which makes gradient-based optimization unsuitable. Under these conditions, an exhaustive search provides a stable and reproducible solution without convergence issues. Although this approach is heuristic, it represents a practical and transparent choice for this targeted industrial application.

Feature-wise normalization is performed using min–max scaling based on the extrema observed in the database for each PCB type. This strategy ensures comparability among heterogeneous feature quantities while preserving their relative variation within the operational data range. Because the database reflects practical industrial conditions, the normalization bounds correspond to realistic feature limits rather than theoretical extremes.

Potential sensitivity to outliers is mitigated in several ways. First, feature values are aggregated at the lot level, which reduces the influence of extreme values from individual images. Second, the weighted similarity formulation allows features that exhibit unstable behavior due to outliers to have a reduced influence through coefficient optimization. While more robust normalization techniques, such as percentile-based scaling or median-based normalization, could further suppress extreme values, the current approach provides sufficient stability and interpretability for the controlled dataset considered in this study.

Since all image features are extracted and stored in advance, the similarity judgment for a given input lot requires only feature normalization and weighted similarity computation. In the experimental environment used in this study, the computation time per similarity query was on the order of several milliseconds, indicating that the proposed method can be applied efficiently in practical industrial workflows.

It should be noted that incorporating more feature types does not necessarily improve image similarity performance. In the proposed method, the five features represent a carefully selected set of complementary descriptors that capture different visual and structural aspects of waste printed circuit boards. These features were not assumed to be equally necessary in all cases; instead, their contributions to similarity judgment were optimized through feature weighting for each PCB type.

This design allows the similarity calculation to emphasize only the most relevant features for a given product category while suppressing redundant or less informative features. As a result, the proposed method achieves robustness and adaptability without relying on an excessive number of features, demonstrating that appropriate feature selection and weighting are more important than simply increasing feature dimensionality.

### 3.4. Comparison Methods

To examine the effectiveness of this approach, the results of the proposed method were compared with those of two comparison methods. The selection of these comparison methods is based on a survey of existing work in related research fields, where two methodological streams are particularly prominent: image similarity and retrieval based on learned feature representations and image-based estimation of physical or material properties using supervised learning. Comparison Method A represents the former stream through self-supervised contrastive learning, which has been widely adopted in recent image similarity and retrieval studies. Comparison Method B represents the latter stream by employing CNN-based regression to estimate quantitative properties from images, a commonly used approach in material analysis and industrial inspection research. Comparison Methods A and B are based on approaches that are widely accepted and commonly used in the computer vision and image analysis community, whereas the proposed method differs from them in how image similarity is defined and computed [41,42]. Comparison Method A evaluates similarity using feature embeddings implicitly learned through self-supervised contrastive learning, a representative framework for image representation learning. Comparison Method B adopts a supervised regression approach that predicts gold content ratios from images, which is a commonly used strategy for estimating quantitative material properties. In contrast to these generally accepted deep learning–based methods, the proposed method explicitly computes image similarity using predefined domain-specific visual and structural features of waste printed circuit boards, including color characteristics, terminal distribution patterns, substrate complexity, and the number of integrated circuits. Unlike the comparison methods, which rely on learned representations and training data, the proposed method does not require training and directly reflects the visual criteria used by human operators in industrial recycling processes.

#### 3.4.1. Comparison Method A

Comparison Method A utilized PCB images as the input to perform feature extraction through self-supervised contrastive learning, followed by gold content prediction via a similarity search. Initially, the dataset was split for each PCB type into training, validation, and test sets in a ratio of 5:1:4. Only PCB types with at least 25 test images were included in the evaluation.

Second, contrastive learning, a type of self-supervised learning, was used to train the model on PCB image features. The parameters utilized during this training process were as follows:

- Model Architecture: ConvNeXtV2 (convnextv2_base);
- Optimization Algorithm: Adam (learning rate: 0.000001);
- Loss Function: NTXentLoss (contrastive loss);
- Image Resizing: 549 × 366 pixels (scaled to 1/10 of original size).

Third, using the trained model, features were extracted from the test PCB images, and similarity searches were conducted. Cosine similarity was utilized for the similarity search, comparing each target image with all images in the database (excluding the target image itself). The 10 most similar PCB images were retrieved based on the cosine similarity scores.

Finally, the gold content ratio of the retrieved similar images was used to calculate the difference ratio relative to the target image, and the prediction success rate was determined accordingly.

#### 3.4.2. Comparison Method B

Comparison Method B estimated the gold content ratio of an input PCB image using a trained model developed from PCB images and their corresponding gold content ratios. Subsequently, the method extracted similar PCB images by identifying those with closely matching predicted gold content ratios.

First, a dataset was constructed for training, following the same procedure as in Comparison Method A. Next, a machine learning regression model was constructed, using EfficientNet as the base network. For Comparison Method B, the dataset was divided into training, validation, and test sets in a ratio of 5:1:4 for each PCB type, following the same data splitting strategy used in Comparison Method A. The parameters utilized during the training process were as follows:

- Model Architecture: EfficientNet (efficientnet_b5);
- Optimization Algorithm: Adam (learning rate: 0.00001);
- Loss Function: MSELoss (mean squared error);
- Image Resizing: 549 × 366 pixels (scaled to 1/10 of original size).

Second, using the model trained for each PCB type, the gold content ratio of each test image was predicted. Based on these predictions, a similarity search was conducted to identify the 10 PCB images with the closest gold content ratios. These images were selected from the entire dataset (i.e., training, validation, and test sets), excluding the target image itself.

Finally, the difference ratio between the predicted gold content of the target image and that of the retrieved similar images was calculated, and the prediction success rate was determined based on this similarity search result.

## 4. Results and Discussion

### 4.1. Evaluation Experiment

To evaluate the accuracy of the similarity determination process, experiments were conducted using 100,000 patterns of feature weight coefficients (described in Section 3.3) on PCBs from the same product lot. The experimental procedure comprised the following four steps:

1. From a group of PCBs belonging to the same product lot, one lot was arbitrarily selected as the target for similarity determination. The image similarity was then calculated between the target lot and all remaining lots.
2. The top 10 lots with the highest image similarity scores were extracted.
3. The differences in the gold content ratio between the target lot and each of the top 10 similar lots (1st: dis1, 2nd: dis2…, 10th: dis10) were calculated. If any one of dis1 through dis10 fell within ±10% of the gold content ratio of the target lot, the similarity determination was successful. The ±10% threshold for gold content difference was determined based on interviews with experienced operators in industrial recycling facilities, who indicated that discrepancies within this range are generally acceptable for practical processing decisions. This criterion therefore reflects operational requirements rather than a statistically optimized tolerance.
4. The role of the target lot was rotated through all remaining lots, and steps 1 through 3 were repeated for each case. The proportion of successful determinations relative to the total number of evaluations was calculated (similarity determination success rate).

### 4.2. Results of Image Similarity Determination Based on the Proposed Method

Table 5 depicts the coefficient values assigned to each image feature when similar judgments were made separately for each product type. The feature weighting coefficients shown in Table 5 differ across PCB types because the visual and structural characteristics that govern similarity judgment are not uniform among product categories. Each PCB type has distinct design features, such as differences in color distribution, terminal arrangement, substrate complexity, and component density. Consequently, the relative importance of the five image features varies depending on the PCB type. For example, the number of integrated circuits has a strong influence on similarity judgment for HDD and memory boards, whereas for other PCB types, features related to color or terminal distribution contribute more significantly. By optimizing feature weights separately for each PCB category, the proposed method emphasizes the most informative features for each board type and avoids the performance degradation that would result from applying a single uniform weighting scheme. Although explicit ablation experiments that remove individual features were not conducted, the PCB-type-dependent feature weighting analysis summarized in Table 5 provides insights into the contribution of each feature to similarity judgment. By observing how the optimized weights vary across PCB types, the relative importance of color, terminal structure, substrate complexity, and IC count can be interpreted. This feature contribution analysis effectively serves as an implicit ablation study, demonstrating how different features influence performance depending on board characteristics. To capture the characteristics of the circuit boards in detail, their external colors and shapes, as well as the types and arrangements of the electronic components mounted on them, should be taken into account. In particular, the number of ICs is closely related to the circuit design and metal content of the board, rendering it an effective feature for image similarity estimation. Therefore, incorporating the number of ICs as a feature likely enables accurate identification of the structural characteristics unique to each board. This feature was particularly significant in cases involving HDD, memory, and modem boards, where it substantially contributed to improved similarity evaluation. The inclusion of the IC count as a feature enhanced the accuracy of the similarity assessment for these product types.

Furthermore, the contribution of each image feature to the similarity computation varies depending on the type of product being considered. Consequently, applying the same set of weighting coefficients across all product types does not improve the identification accuracy. Accordingly, product-specific feature weighting coefficients were optimized for the image similarity calculation process.

**Figure 9:**
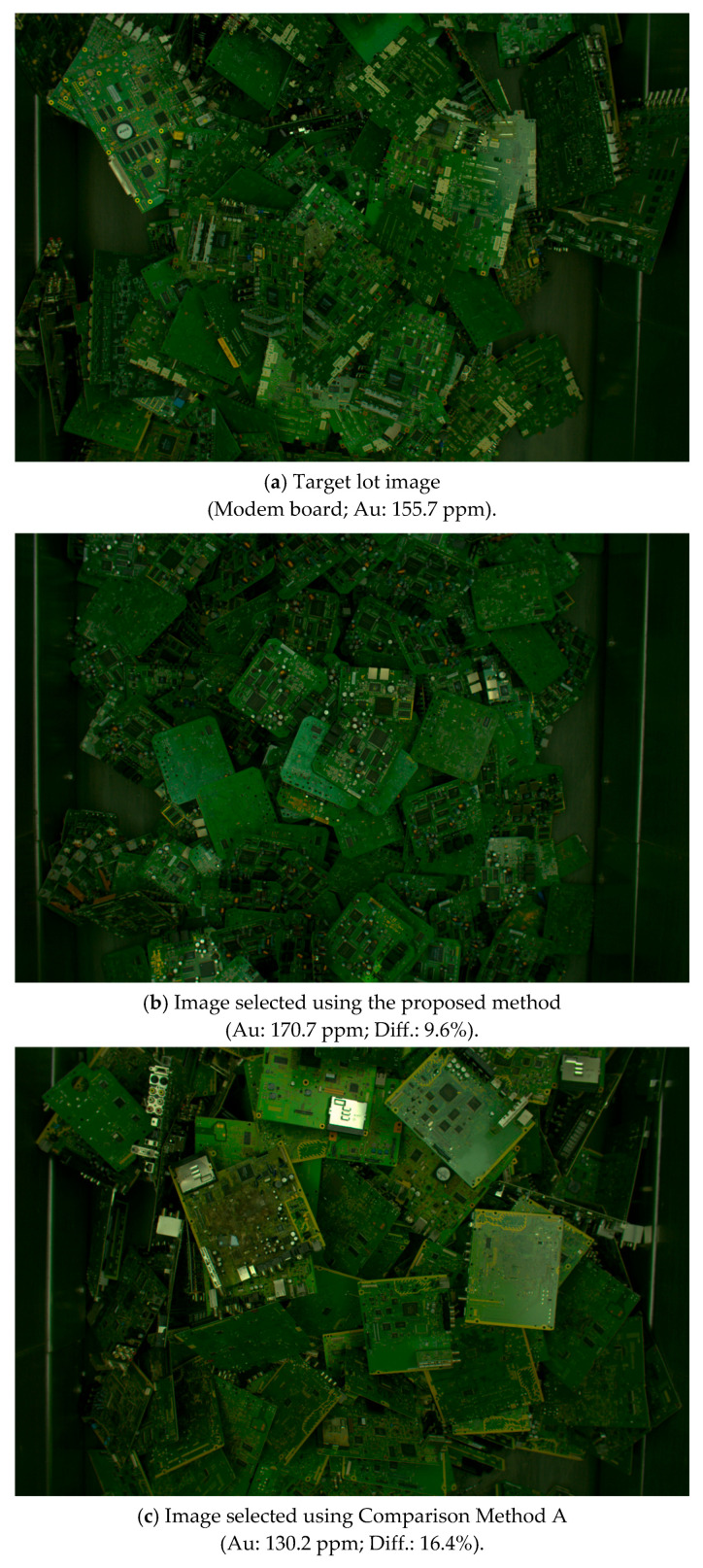

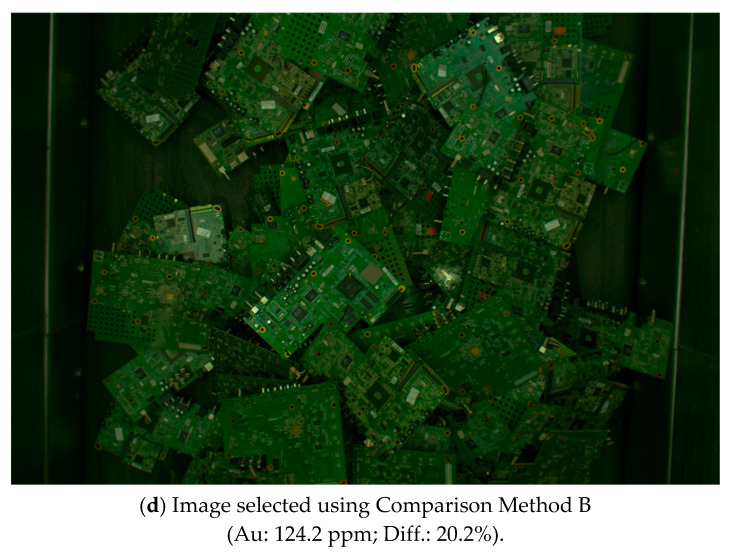
Example of image similarity calculation results for modem boards (2/2).

Table 6 lists the classification accuracies achieved by each comparison method. Figure 9 shows an example of image similarity calculation results for the modem board. For each method, the lot with the smallest difference in gold content among the top 10 lots ranked by image similarity was selected. In Figure 9, the notation “Au: xxx ppm” represents the measured gold content ratio of the PCB lot selected as similar to the target lot, while “Diff.: yyy%” denotes the relative difference between the gold content of the selected lot and that of the target lot. The difference is calculated as the absolute percentage deviation with respect to the target lot’s gold content. According to the evaluation criterion defined in this study, a similarity judgment is considered successful when at least one of the top ten selected lots exhibits a gold content difference within ±10%. Therefore, a difference of 16.4%, as shown in Figure 9c, indicates that the comparison method failed to identify a sufficiently similar lot.

The proposed method outperformed Comparison Method A by 2.5% in terms of the classification accuracy across all types of boards. In particular, for frame boards, the classification accuracy of the proposed method was 12.5% higher than that of Comparison Method A. These results indicate that the proposed method could accurately determine image similarity for the boards targeted in this study. In contrast, for modem boards, the proposed method achieved a lower result of 80.0%, lower than those for the other types of boards. This phenomenon could be attributed to the complex and diverse surface structure of modem boards, which causes features to be widely dispersed. Therefore, grouping similar boards within the modem category and calculating the image similarity for each classified group could improve the accuracy.

Compared with Comparison Method B, the proposed method exhibited an improvement of 56.1% in classification accuracy across all types of boards. One possible factor contributing to the lower accuracy of Comparison Method B involves the limited number of training data available for certain PCB types. Because this method relies on supervised regression to predict gold content ratios from images, its performance is inherently influenced by the size and distribution of the training dataset. Rather than indicating an inherent limitation of the method itself, this result highlights the practical challenges of applying training-dependent models in real-world WPCB recycling environments, where acquiring large, well-balanced labeled datasets is often difficult. Comparison method B utilized a neural network based on EfficientNet, with board images and the gold content ratio as inputs for training. The assumption was that the model was not sufficiently trained. In contrast, the proposed method calculates the image similarity without requiring training and operates independently of the number of training data. Additionally, although the boards in this study were of the same type, they had diverse gold contents. Therefore, in Comparison Method B, where the gold content ratio of the entire board image was used as the input for training, handling the feature variations in individual boards within board images was difficult. These results indicate that for the data of interest in this study, the proposed method is more useful than Comparison Method B for calculating image similarity.

Although the individual image features considered in this study, such as color information or structural descriptors, have been explored in previous image analysis research, the novelty of the proposed method lies in their domain-specific integration and application to WPCB similarity judgment. In particular, the proposed approach combines multiple interpretable visual and structural features that are closely related to the physical composition and appearance of WPCBs and evaluates similarity at the lot level, which reflects actual industrial recycling workflows.

In contrast to deep learning-based methods that implicitly learn image representations and require large amounts of training data, the proposed method operates without a training phase and provides explicit control over feature contributions through optimized weighting coefficients. This design enables stable and robust similarity judgment even under limited-data conditions and allows the similarity assessment process to remain interpretable. As a result, the proposed method offers practical advantages in terms of reliability, transparency, and applicability to real-world WPCB recycling environments, distinguishing it from existing image similarity approaches.

It should also be noted that the scope of the comparative experiments in this study is intentionally focused. The primary objective is not to exhaustively benchmark the proposed method against all possible traditional or learning-based similarity measures but to evaluate its effectiveness under realistic industrial conditions using representative and widely accepted baselines. Many conventional image similarity methods and public benchmark datasets are designed for single-object images captured under controlled environments, which differ substantially from the lot-based WPCB images used in this study.

Within this constrained but practical setting, the comparative results highlight fundamental differences in applicability between training-dependent approaches and the proposed training-free framework. In particular, the analysis indicates that methods that rely on learned representations are sensitive to data availability and distribution, whereas the proposed method benefits from explicit domain knowledge and interpretable feature integration. Comprehensive evaluation using additional traditional methods and public datasets remains an important direction for future work, especially as suitable datasets that reflect industrial WPCB recycling conditions become available.

The proposed method is designed as a domain-specific image similarity judgment framework for WPCB recycling and is not intended to be universally applicable without adaptation. It could be feasibly generalized to unseen PCB types within the same recycling facility by recalibrating feature weighting coefficients to the new PCB category, as the underlying feature definitions remain applicable. This process requires only offline optimization and does not alter the runtime similarity computation. Generalization across different imaging setups or recycling plants may be affected by changes in imaging conditions, board handling processes, and material characteristics. Although the current study uses controlled darkroom imaging, applying the method to environments with different illumination or camera configurations may require adjustment to normalization ranges or feature weighting. Furthermore, the present evaluation is limited to ten PCB types obtained from a single industrial context, which may not capture the full diversity of WPCBs encountered globally. These limitations highlight important directions for future work, including validation on unseen PCB types, cross-facility evaluation, and adaptation to less controlled imaging environments. Despite these constraints, the proposed framework provides a flexible and interpretable foundation for similarity judgment that can be systematically adapted to new recycling scenarios.

Regarding the dataset scale and image acquisition conditions, the experiments were conducted using multiple lots for each of the ten PCB types shown in Table 1, resulting in a total of several hundred lot images being evaluated in this study. Although the number of PCB categories is limited, the proposed method does not rely on learning PCB-specific patterns through model training. Instead, similarity judgment is performed using predefined, interpretable features aggregated at the lot level, which reduces sensitivity to individual board styles and mitigates the risk of overfitting.

All images were acquired in a controlled darkroom environment with fixed camera, illumination, and camera-to-board distance settings. External light was completely blocked, and imaging conditions were kept constant throughout data acquisition. As a result, the influence of camera-specific artifacts and illumination variations on color- and structure-based features was minimized. While expanding the evaluation to additional PCB types and acquisition setups remains an important topic for future work, the current results demonstrate that the proposed approach provides stable and reliable similarity judgment under realistic and well-controlled industrial imaging conditions.

It should be noted that robustness to large illumination changes was not explicitly evaluated in this study, as all images were acquired under controlled darkroom conditions with fixed lighting and camera settings. Within this controlled environment, the potential sensitivity of hue-based features was mitigated through HSV color space conversion, overlapping hue binning, and lot-level feature aggregation, which together suppress minor residual variations.

Systematic evaluation of the proposed method under intentionally varied illumination conditions would provide additional insights into robustness and has been identified as an important topic for future work, particularly for applications involving less controlled imaging environments.

## 5. Conclusions

In this study, a method for calculating image features and similarity from input PCB images was proposed, and the accuracy of similarity determination based on PCB characteristics through classification was evaluated. The following conclusions can be drawn from the results:

- The following features extracted from PCB images are effective in image similarity assessment: hue values, coefficients of variation focused on terminal regions, numbers of linear elements in terminal regions, structural complexity, and numbers of ICs.
- Applying weightings to each feature based on its respective contributions improves the success rate of the similarity assessment.
- The proposed image similarity judgment method achieved an accuracy of 88.0% for the PCB types targeted in this study and outperformed a comparative method using self-supervised contrastive learning for feature extraction in terms of similarity judgment accuracy.

These results demonstrate that incorporating domain-specific features and optimized weighting strategies can substantially improve the accuracy of similarity determination relative to existing methods. Furthermore, the proposed approach reduces reliance on manual inspection, thereby lowering operational costs and minimizing human error in industrial recycling environments.

For future work, the range of target PCB types should be expanded to assess the generalizability of the proposed method. In particular, the judgment accuracy for communication boards, motherboards, modem boards, and frame boards was below 90.0%. To address this, a stepwise evaluation strategy should be considered. For example, pre-classifying motherboards into desktop and laptop categories before performing similarity calculations may improve the accuracy. Additionally, integrating advanced deep learning techniques into domain-specific feature engineering could further enhance performance and scalability.

## Figures and Tables

**Table 1:** Number of lots for each board type.

PCB Type	Number of Lots
HDD boards	65
Mobile phone boards	105
Communication boards	345
Laptop PC boards	71
Motherboards	218
Memory boards	76
Modem boards	84
Frame boards	87

**Table 2:** Specifications of the camera used.

	Specification
Camera model	Basler acA5472-17uc
Image resolution	5496 × 3672 pixels
Lens model	Basler C-11-1620-12M-P
Mount type	C-mount
Focal length	16.0 mm
F-number (aperture)	f/2.0–16.0
Floating mechanism	None

**Table 3:** Correspondence between hue classifications and hue values.

Hue Classification	Hue Values	Hue Classification	Hue Values
Class 1	345–360, 0–4	Class 13	165–184
Class 2	0–19	Class 14	180–199
Class 3	15–34	Class 15	195–214
Class 4	30–49	Class 16	210–229
Class 5	45–64	Class 17	225–244
Class 6	60–79	Class 18	240–259
Class 7	75–94	Class 19	255–274
Class 8	90–109	Class 20	270–289
Class 9	105–124	Class 21	285–304
Class 10	120–139	Class 22	300–319
Class 11	135–154	Class 23	315–334
Class 12	150–169	Class 24	330–349

**Table 4:** HSV ranges at terminal sections.

	Values
Hue	32–82
Saturation	20–100
Value	30–100

**Table 5:** Coefficient values assigned to each image feature.

PCB Type	α	β	γ	δ	ε
HDD boards	0.1	0.7	0.0	0.3	0.4
Mobile phone boards	0.6	0.0	0.1	0.7	0.2
Communication boards	0.6	0.0	0.0	0.3	0.0
Laptop PC boards	0.0	0.6	0.6	0.4	0.0
Motherboards	0.6	0.8	0.0	0.1	0.1
Memory boards	0.5	0.5	0.5	0.4	0.5
Modem boards	0.0	0.0	0.7	0.4	0.5
Frame boards	0.0	0.7	0.4	0.0	0.0

**Table 6:** Success rate of judgment for each method.

PCB type	Proposed Method	Comparison Method A	Comparison Method B
HDD boards	96.2	96.2	65.4
Mobile phone boards	97.6	92.9	59.5
Communication boards	82.0	81.2	19.7
Laptop PC boards	96.6	93.1	31.0
Motherboards	87.1	87.1	5.9
Memory boards	96.8	96.8	77.4
Modem boards	80.0	75.0	37.5
Frame boards	87.5	75.0	34.4

## Data Availability

The PCB image data and associated metal composition information used in this study were provided by industrial recycling facilities and are subject to confidentiality restrictions. Therefore, the raw data cannot be made publicly available. Detailed descriptions of the proposed algorithm, feature extraction procedures, and parameter settings are provided in the manuscript to support reproducibility.

## Abbreviations

The following abbreviations are used in this manuscript:

e-waste	Electronic Waste
WPCBs	Waste Printed Circuit Boards
YOLO	You Only Look Once

## Appendix A

This appendix provides the pseudo-code of the proposed image similarity judgment method to facilitate understanding of the computational flow described in Section 3. The pseudo-code summarizes the sequence of feature extraction, normalization, weighting, and similarity calculation steps based on the mathematical formulations presented in the main text.

**Algorithm A1**: Image Similarity Judgment for WPCB Lots
Input: $L_{q}$: Input PCB lot $\left\{L_{d}\right\}$: Set of database PCB lots of the same PCB type α, β, γ, δ, ε: Feature weighting coefficients ** ** Output: Ranked list of database PCB lots according to similarity ** ** 1: Extract hue-based feature $F_{c}$ from $L_{q}$ and each $L_{d}$ 2: Extract terminal-region variation feature $F_{v}$ from $L_{q}$ and each $L_{d}$ 3: Extract terminal line feature $F_{L}$ from $L_{q}$ and each $L_{d}$ 4: Extract substrate complexity feature $F_{s}$ from $L_{q}$ and each $L_{d}$ 5: Extract IC count feature $F_{I}$ from $L_{q}$ and each $L_{d}$ 6: For each database lot $L_{d}$ do 7: Compute normalized feature-wise similarity scores 8: Compute overall similarity score:
$S=\left(\alpha F_{c}+\beta F_{v}+\gamma F_{L}+\delta F_{s}+\varepsilon F_{I}\right)/\left(\alpha +\beta +\gamma +\delta +\varepsilon \right)$
9: End for 10: Rank all database lots in descending order of S 11: Output the ranked list
